# Supplementary material for: The Future Packaging of the Food Industry: The Development and Characterization of Innovative Biobased Materials with Essential Oils Added
Source: Gels. 2022 Aug 14;8(8):505. doi: 10.3390/gels8080505 (PMC9407569; doi:10.3390/gels8080505)
Supplement: Supplementary file 1 [file gels-08-00505-s001.zip › gels-1855949-supplementary.pdf]

The Future Packaging of the Food Industry: The Development and Characterization of Innovative Biobased Materials with Essential Oils Added

Roxana Gheorghita Puscaselu [1], Andrei Lobiuc [1,*] and Gheorghe Gutt [2]

[1] Faculty of Medicine and Biological Sciences, Stefan Cel Mare University of Suceava, 720229 Suceava, Romania
[2] Faculty of Food Engineering, Stefan Cel Mare University of Suceava, 720229 Suceava, Romania
* Correspondence: andrei.lobiuc@usm.ro

Figure S1. Photos of the films, the regular edges and the homogeneity of the films can be observed

B1 – 10% lemon EO	***B2 – 20% lemon EO***	***B3 – 10% grapefruit EO***	***B4–20% grapefruit EO***
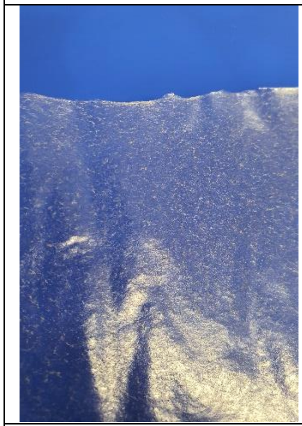	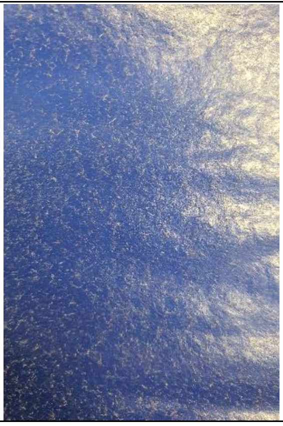	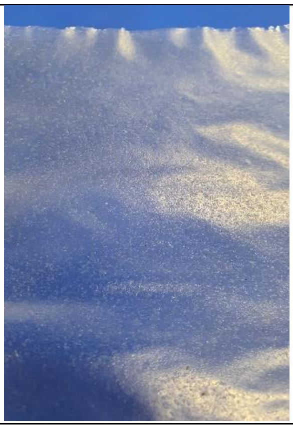	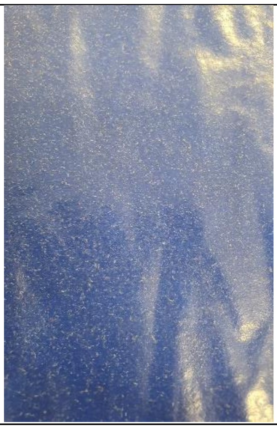
B5 – 10% lemon EO	***B6 – 20% lemon EO***	***B7 – 10% cinnamon EO***	***B8 – 20% cinnamon EO***
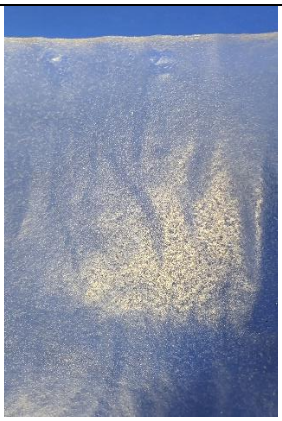	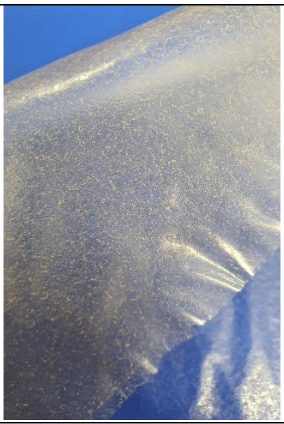	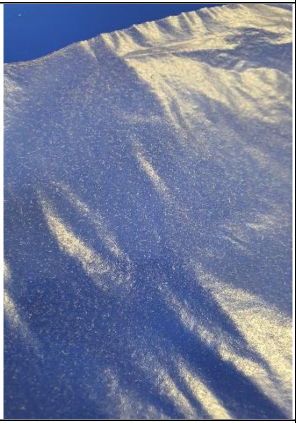	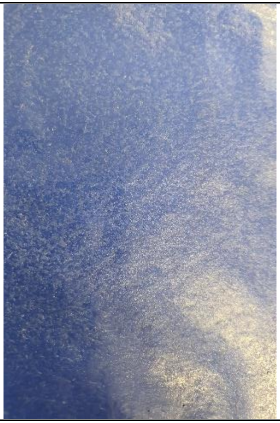
B9 – 10% clove EO	***B10 – 20% clove EO***	***B11 – 10% mint EO***	***B12 – 20% mint EO***

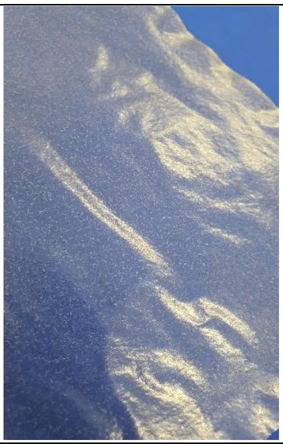	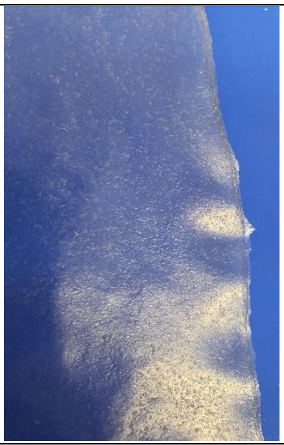	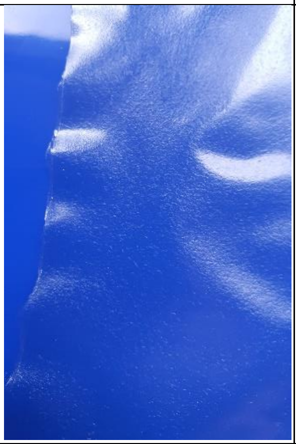	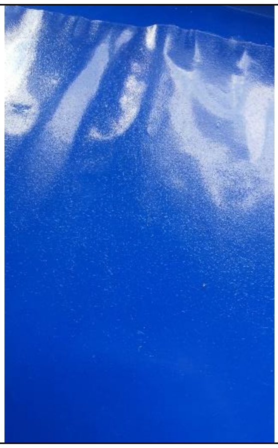
B13 – 10% chamomile EO	***B14 – 20% chamomile EO***	***B15- 10% ginger EO***	***B16 – 20% ginger EO***
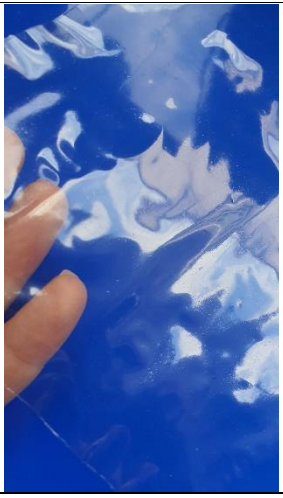	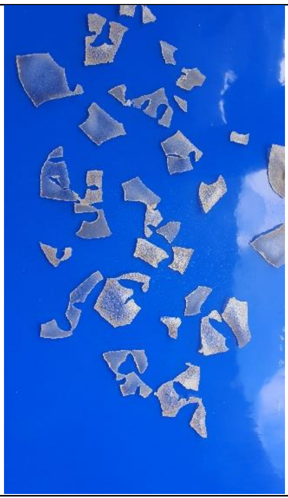	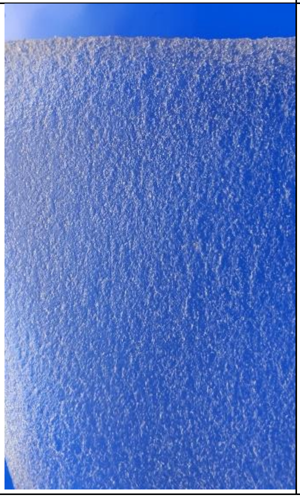	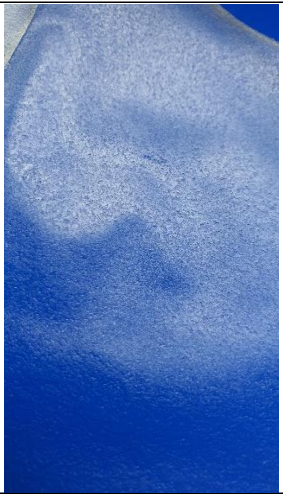
B17 – 10% eucalyptus EO	***B18 – 20% eucalyptus EO***	***B19 – control, no EO added***	
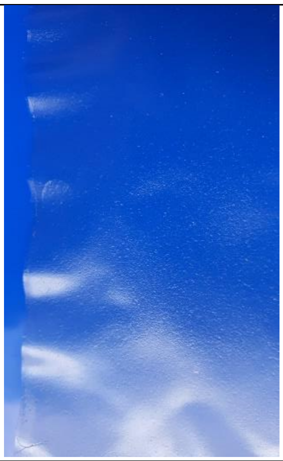	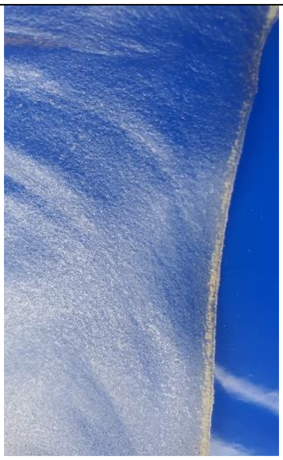	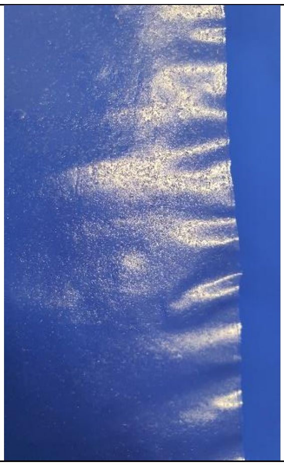	
